# Motor Development among Spanish Preschool Children

**DOI:** 10.3390/children8010041

**Published:** 2021-01-12

**Authors:** Cristina Honrubia-Montesinos, Pedro Gil-Madrona, Luisa Losada-Puente

**Affiliations:** 1 Faculty of Education of Albacete, University of Castilla-La Mancha, 02001 Albacete, Spain; Pedro.Gil@uclm.es; 2 Faculty of Education, University of A Coruña, 15071 A Coruña, Spain; luisa.losada@udc.es

**Keywords:** motor development, locomotor skills, object control skills, age, sex, only child, prematurity

## Abstract

The purpose of this study was to analyze motor development of Spanish preschoolers, taking into account sex and age, being an only child, prematurity, and the practice of extracurricular activities. The sample was composed of 300 preschoolers (132 girls, 168 boys) ages 3 to 6 years. Preschoolers were tested on 12 fundamental motor skills (locomotor and object control) through the Test of Gross Motor Development—Second Edition (TGMD-2). Nonparametric analysis indicated that there are differences between girls and boys in locomotor and object control skills in the age range of 3–4 years. However, boys and girls scored similarly at the age of 5 years in locomotor development. There were not differences between only children and those who are not only children. Similarly, prematurity was not associated with locomotor and object control development. Nevertheless, those preschoolers who practice extracurricular physical activities scored significantly higher in comparison with those children do not. Further research is needed to shed light on the differences between boys and girls in object control. It may be explained by the types of extracurricular activities.

## 1. Introduction

Fundamental movement skills (FMS) have been described as the building blocks for movement and form the foundation for many of the specialized movement skills needed to participate successfully in sport and physical activity [1]. As FMS do not generally develop naturally, they need to be learned, practiced, and developed [2]. Childhood is a critical time for FMS development as recent reviews have found FMS proficiency to be positively associated with a range of health, fitness, and academic outcomes, participation in organized sports, and sustained engagement in physical activity [3,4,5]. Despite knowing that these skills play a crucial role in health and physical activity habits, studies have shown that preschool children present motor delays and during this stage an increasing number of young children have insufficiently developed FMS [6].

The acquisition of FMS is not only achieved through natural development and maturation, but also through continuous interaction with a stimulating and supportive social and physical environment. In other words, FMS must be instructed and promoted [7,8]. For this reason, and according to a socio-ecological approach, the child’s environment plays a vital role in motor development. This concept is based on a mutual interaction between the biological conditions and the environment that can be seen as a dynamic developmental system of perception and action [1,9].

Starting with the gender, the study developed by Goodway et al. [10] with 469 preschoolers concluded that no differences were found between boys and girls in locomotor skills, but boys scored higher in object control. Furthermore, Bardid et al. [11], with a sample of 1614 children aged 3–8 years old, found that boys performed better than girls in object control in all age groups, whereas no differences were discovered in locomotor development. Similarly, Kokštejn et al. [12] concluded that, at the ages of 3, 4, and 5 years, no differences were observed in the control of objects. However, at age 6 boys outperformed girls in controlling objects. In the same way, the study by Saraiva et al. [13], with preschoolers of 3, 4, and 5 years of age in whom they found differences in the control of objects, they persisted in all age groups, favorable to boys. However, this research concluded that there were no differences in development of locomotor skills between boys and girls in any age range. Focusing the attention on Spanish children, Amador-Ruiz et al. [14] measured motor development using Movement Assessment Battery for Children (2nd ed.) and found that boys scored significantly higher in control of objects but girls did it in locomotor skills. No previous studies have been found measuring differences between boys and girls in motor development through the Test of Gross Motor Development (TGMD-2). It seems that, according to these previous studies [11,12,13,14], these differences are not related to biological factors but they are explained by environmental variables. Following with the Spanish context, boys tend to choose activities more related to object control, while girls seem not to choose these kind of activities [15]. It could be a plausible explanation for the existence of differences in object control.

Another determining factor in motor development is prematurity. According to the World Health Organization [16], a child is considered premature when a baby is born before 37 weeks of pregnancy. Temple et al. [17] evaluated the motor development of premature and non-premature children, not finding significant differences regarding their effect on the control of objects and/or locomotor development. In addition, these authors added that motor skills at the beginning of the Early Childhood Education (ECE) stage present similar levels with respect to those students who were born prematurely. Also, Santos et al. [18] found that healthy premature infants do not experience profound delays in motor competence.

On the contrary, Bruininks and Bruininks [19] measured motor development by Bruininks–Oseretsky Test of Motor Proficiency (2nd ed.) with 8–9-year-old children and concluded that premature children have worse motor development compared to full-term infants. Similar results were found by Prins, Von Lindern, Van Dijk, and Versteegh [20], who revealed that the preterm infants scored significantly lower in comparison with those born full term and these differences are persistent through time. Thus, according to Rizzardo and Bredin [21], the detection of motor delays in preterm children can allow schools to design and implement early measures to alleviate these delays.

Continuing with environmental factors and more specifically with the fact of being an only child, different studies have concluded that having an older sibling is a determinant of motor development due to the fact that he/she acts as a model to reproduce the motor behavior [22,23]. Thus, the order of birth in a family is also a factor that can affect the development of motor skills. Children with older siblings have better motor performance than only or first-born children [24,25]. One of the typical sequences is found to be that an older child initially performs a task while younger siblings watch or spend a lot of time observing the older sibling’s performance and replicating the movements [26,27]. Older siblings provide more advanced developmental models for younger siblings and help create an enriched and stimulating environment that appears to enhance the development of younger siblings [20,25]. More recently, Giagazoglou et al. [28] analyzed motor development according to the order of birth occupied by the child and concluded that said order did not have an influence on motor development.

Another factor, within those of the environmental ones, is the extracurricular activity outside of school hours. Numerous investigations have concluded that physical activity impact positively on FMS [29,30,31,32]. The study carried out by Temple et al. [33] corroborated that the more active extracurricular activities were associated with a higher level of motor skills. In the same way, Burns et al. [34] revealed that moderate to vigorous physical activity was significantly associated with higher TGMD-2 total scores. Taking into account the Spanish context, different investigations [35,36,37] concluded the importance that children aged 3–6 years old practice extracurricular activities due to the fact that it was associated with higher scores in motor development.

Bearing in mind what has been explained above but also the lack of investigations with Spanish children exploring motor development, the objective was to analyze Spanish preschoolers’ motor development taking into account sex, age, being or not an only child, prematurity, and the participation in extracurricular physical activities.

## 2. Materials and Methods

The methodology used for this present study was quantitative, through a cross-sectional and observational design, where data collection was carried out at a specific time, allowing for the establishment of cause–effect relationships between them [38].

Three hundred children from Spain (*n =* 132 girls and *n =* 168 boys) took part in this study. Their ages ranged from 3 to 5 years old: 36.6% (*n* = 109) were 3 years old, 38.7% (*n* = 116) were 4 years old, and, finally, 24.9% (*n* = 75) were 5 years old. In relation to their sibling history, 40.7% (*n* = 122) of preschoolers were only children and 59.3% (*n* = 178) were not only children. Regarding prematurity, as can be seen in Table 1, only 7% (*n* = 21) of the infants were premature, whereas 93% (*n* = 279) were full-term babies. In relation to extracurricular activity (Table 1), there was a homogeneous distribution of the sample, where 58% (*n* = 174) of the participants did carry out some type of activity outside the classroom and 42% (*n* = 126) did not currently carry out activities of an extracurricular character. Thus, the present sample can be defined in the following terms: non-probabilistic and convenience. Being enrolled in the Early Childhood Education stage was the main criterion to select the participants.

The Test of Gross Motor Development-Second Edition (TGMD-2) is a standardized, criterion- and normative-referenced, valid, and reliable gross motor assessment for children aged 3–10 years and 11 months [39]. The TGMD-2 measures 12 motor skills across two subscales: locomotor (run, gallop, hop, leap, jump, and slide) and object control (throw, catch, kick, strike, roll, and dribble) skills. Children complete one practice trial and two scored trials for each skill. Each skill possesses a range from 6 to 10 points depending on the number of critical elements. The scores for each skill within a subscale are then summed for a raw skill subscale score. Each subscale can be combined for an overall gross motor raw skill score. Raw scores for each subscale or for overall gross motor can be converted into standard scores and percentile ranks (based on age and sex) to serve as a normative reference valid in a variety of countries (e.g., United States, Brazil, and Germany). The standard scores from each subscale can be combined and converted into an overall gross motor quotient, which is also a normative reference for valid populations.

The first step that was carried out was to contact the educational institutions to inform about the purpose of the research. Then, in those centers that agreed to participate, meetings were held with the parents to explain the study in detail. Finally, an obtained written informed consent of motor development of preschoolers was assessed by the first author of this research. It was developed, taking into account the manual provided by TGMD-2 [39]. This study was approved by Ethics Committee of the University of Castilla La Mancha.

All TGMD-2 trials were conducted in the indoor sports hall of the participating schools. To avoid any distraction, no other physical education or sports lessons were conducted in the hall during the Fundamental Motor Skills testing sessions. The assessment was developed following the manual and modifications were not needed. Following an accurate demonstration and verbal description by one of two trained researchers and one practice trial, a child was given two trials for each skill. Both trials were video recorded, and each child’s skill rating was based on the video analysis by the same researchers. Each skill had a set of three to five performance criteria, and the child’s performance was assessed using a score of 0 or 1 for each performance criterion in each trial. If the skill criterion was adequately demonstrated, one mark would be given, while a zero mark was given if the participant failed to sufficiently demonstrate the skill criterion.

The information about prematurity, being only child, and extracurricular activities was provided by families. Parents completed the informed consented in which they were required to provide data of their children. They answered whether or not their children take part in any extracurricular physical activity, are only children, or preterm babies.

With respect to data analysis, it was performed with the Statistical Package for Social Sciences (SPSS) 25. In the first place, it was found that the sample was not distributed according to normality, so nonparametric statistics were used. Specifically, the Kruskal–Wall H and Mann–Whitney U tests were used to compare the different groups, adopting a significance criterion of *p* < 0.05. Furthermore, omnibus tests were performed to analyze the influence of age and gender in motor development. To do that, the standardized scores were used as dependent variables in which percentiles below 50 were considered low and percentiles above 50 were high. The effect size was calculated through Cohen’s d, which was interpreted as follows: small effect when *d* = 0.20, medium when *d* = 0.50, and high when *d* = 0.80.

## 3. Results

The results are going to be presented taking into account sex, age, being or not an only child, prematurity, and the practice of extracurricular activities.

Starting with the performance criteria in locomotor skills, Table 2 shows that the lowest mean for both boys and girls was the component 5 in the hop, whereas the highest means for boys and girls were the components included in the skills gallop, components 1 and 2, respectively. The skill that scored the highest was the horizontal jump for girls; however, for boys it was the hop. With respect to the mastery of each criterion, Table 3 shows that the lowest percentage of children who demonstrated mastery is for the component 4 in the gallop and the component 2 in the horizontal jump. The highest proportion of children was for the components that compose the run.

In relation to object control skills, Table 4 shows that the lowest mean for both genders was the component 4 in the dribble, whereas the highest mean for both boys and girls was the component 1 in the kick. The skill that obtained the highest mean for girls was the dribble. On the other hand, the skill with the highest score was the kick. Regarding the mastery of each criterion (Table 5), in this case, the lowest proportion was for the component 4 in the dribble, but the highest percentage of children who demonstrated mastery was the component 1 in the kick.

Comparing Spanish with American children, as can be seen in Table 6, the proportion of children who demonstrated mastery was higher in the subtest of locomotor skills and this proportion was similar to American children. Furthermore, the proportion of Spanish children who demonstrated mastery of object control was smaller compared to American children.

With reference to the age and gender and starting with the age of 3 years (Table 7), there was a difference of means between boys and girls of 1.38 in locomotor development and 3.19 points in object control. As well, statistically significant differences were found both in locomotor development (*p* = 0.030, *d* = 0.08) and object control (*p* = 0.003, *d* = 0.43) in favor of boys. In relation to 4-year-old children, boys continue to obtain a higher average in locomotor development, with a difference of 3.44 points compared to girls and, above all, in control of objects (difference of 4.3 points). Both differences were statistically significant in locomotor (*p* = 0.004, *d* = 0.48) and object control development *(p* = 0.003, *d* = 0.42), with a medium effect size in both cases.

At the age of 5 years, as can be observed in Table 7, again the boys had higher scores. In this case, the difference of mean was reduced in locomotor development (1.15 points). However, it was increased in object control up to 5.43. Only in this last domain (object control) were the differences statistically significant (*p* = 0.003, *d* = 0.50). As can be seen in Table 7, regarding locomotor development (*p* = 0.233, *d* = 0.36), no differences were found between boys and girls at that age.

The dependent variable locomotor development was dichotomous. The predictors were the age and gender. The objective of each study was to predict or to distinguish the outcome categories on the basis of predictors. In this model, χ^2^ = 8.5 (*p* < 0.005) indicated a good model fit. R^2^ indices showed that 2.8% of the total variance was explained by age and gender. The β values (β = 0.256, 0.014) showed that independent variables influenced dependent variable. The odds of age for presenting higher locomotor development were 1.04 (= e^014^) times greater than those children who were younger. However, the model was not significant for object control development (*p* = 0.234).

With respect to whether or not preschoolers are only children, Table 8 presents the values of central tendency (mean and median) and dispersion (standard deviation) of locomotor development and object control as well as the Mann’s U test. As can be observed, there was a difference of means of only 0.04 points in locomotor development. It increased in object control (1.01 points). Significant differences between only children and non-only children were not found.

Continuing with the variable extracurricular activity, Table 9 shows the proportion of children who demonstrated mastery, taking into account the participation in extracurricular physical activities. In respect to locomotor skills, we found the prevalence of mastery was higher among those who practiced extracurricular physical activities. These differences were higher for the run and gallop. Regarding object control skills, the proportion of children who demonstrated mastery was higher in those who develop extracurricular activities. These differences of percentages were higher in the catch and kick.

Observing Table 10, there was a difference of means of 1.75 points in locomotor development in favor of those who did it. This difference increased up to 5.84 points in object control. The Mann-Whitney U test (Table 10) allowed us to verify the presence of statistically significant differences in favor of those who carry out extracurricular activities in object control development (*p* < 0.001), although with a small effect size (*d* = 0.20). On the contrary, there were no significant differences regarding locomotor development (*p* = 0.60). Thus, those preschoolers who practiced extracurricular activities showed significantly higher scores in object control.

Finally, taking into account prematurity (Table 11), the differences of means in locomotor and object control skills were 3.42 and 2.43, respectively. The Mann–Whitney U test did not show differences in either locomotor or object control development between preterm and full-term children.

## 4. Discussion

The main aim of this research was to analyze Spanish preschoolers’ motor development, taking into account sex, age, being or not an only child, prematurity, and the practice of extracurricular activities.

In relation to the comparison between boys and girls, we revealed the existence of significant differences at the ages of 3 and 4 years old in locomotor and object control. In both cases, boys scored significantly higher than girls. Furthermore, no statistically significant differences in locomotor development were found at the age of 5 years. However, these differences were still found in object control. These data partially agree with those obtained in the study by Saraiva et al. [13] with preschoolers of 3, 4, and 5 years of age in whom they found differences in the control of objects: They persisted in all age groups, favorable to boys. However, this research concluded that there were no differences in development of locomotor skills between boys and girls in any age range. Hardy et al. [3], with a sample of 425 children between 4 years and 4.9 months, found significant differences in locomotor development or control of objects. On the other hand, the research carried out by Pienaar, Van Reenen, and Weber [40], with a sample of 109 6-year-old children, found no differences in locomotor development, but did in the control of objects. These findings, according to previous studies [13,41], are not explained by biological factors due to the fact that boys and girls are very similar physically [8] (body type, body composition, strength, and limb lengths). For this reason, gender differences in object control development are more likely to be influenced by a child’s environment such as families, peers, and teachers [42,43].

Continuing with the differences between Spanish and American preschool children, Spanish children had lower levels of motor competence than the US reference sample, specifically for object control skills. This finding is consistent with the study developed by Bardid et al. [44], in which a Belgian sample scored worse on object control skills. An explanation, according to these authors [44], may be the cultural differences between children from different countries. As well, it could be explained by the secular decrease in motor competence [44].

Taking into account being or not an only child, we confirmed the independence of motor development and the fact of being or not being an only child due to the fact that it has no statistically significant differences. A plausible explanation for this finding could be that there are other variables related to the parental environment that affect motor development [42]. Therefore, the result obtained confronts previous findings, such as that of Krombholz [25] about the influence of the older sibling as a model in motor performance.

Regarding the performance of extracurricular activities, it was found that the practice of extracurricular activities has a positive influence on object control. Furthermore, the percentage of children who demonstrated mastery was higher in comparison with those do not practice extracurricular physical activities These findings are consistent with numerous studies that have shown the impact of physical activity on motor development [29,30,31,32]. As well, those results obtained by Temple et al. [33] highlighted that active extracurricular activities promote motor skills. Additionally, these authors suggested that the performance of these activities leads to the improvement of motor development. As well, the research carried out by Suen et al. [45], in which they analyzed the impact of family factors on motor development in preschoolers, revealed that intrinsic infant variables such as age and participation in moderate to vigorous physical activity are the most effective predictors of motor development. Likewise, a study by Skowroński et al. [46], where the hours of physical education were increased at the extracurricular level, caused a significant development both in locomotor development and in object control. For this reason, and taking into account the literature, it is of utmost importance the practice of extracurricular physical activities as they may seem to be a perfect vehicle to the promotion of FMS.

Continuing with prematurity, no significant differences were found between preterm and full-term preschoolers in both locomotor and object control. The findings are congruent with the research carried out by Temple et al. [17], due to the fact that they observed that prematurity does not exert an effect on object control or locomotor development since both premature infants and non-premature infants scored similarly at the beginning of ECE stage. In the same way, Raniero, Tudella, and Mattos [47] concluded that healthy premature infants did not present profound delays in motor competence. On the contrary, some studies have evidenced different findings. Bruininks and Bruininks [19] corroborated that at the ages of 8 and 9, children born prematurely presented a lower level compared to term children. Prins and colleagues [20] highlighted that these motor deficits are persistent over time. In this sense, Rizzardo and Bredin [21] stressed that the detection of motor delays in premature children could improve motor development since it would allow schools to design an early implementation of measures to alleviate these delays.

## 5. Conclusions

This study shed light on what factors influence motor development. It was revealed that prematurity and being an only child are not determinants. However, extracurricular activities where children practice physical activity might have a positive impact on motor development. With respect to sex, further research is needed to understand the existence of differences in object control in preschoolers. Taking into account the findings of the study, it is of utmost importance highlighting that no differences between boys and girls were found at the age of 5 years in locomotor development. A plausible explanation of this finding may be found in the practices developed in ECE. However, it is necessary to implement more physical education sessions in which object control skills can be worked to reduce the differences between boys and girls. On the other hand, it was found that those who perform extracurricular activities scored significantly higher. It may explain the differences between boys and girls in object control skills. For this reason, the impact of the practices developed in early childhood on motor development requires further research. As well, another future line of research may be the analysis of extracurricular activities chosen by boys and girls in this stage.

## Figures and Tables

**Table 1:** Descriptive statistics of the sample.

	Prematurity		Being an Only Child		Extracurricular Activity	
	N	%	N	%	N	%
Yes	21	7	122	40.7	174	58
No	279	93	178	59.3	126	42

**Table 2:** Means (M) and standard deviations (SD) of performance on the Test of Gross Motor Development (2nd version) TGMD-2—item scores and subtest scores—for boys and girls in locomotor development.

	Girls	Boys
	M (SD)	M (SD)
Run	4.32 (1.77)	4.47 (1.75)
Arms move in opposition to legs, elbows bent	1.13 (0.65)	1.23 (0.70)
Brief period where both feet are off the ground	1.29 (0.78)	1.32 (0.66)
Narrow foot placement, landing on heel or toe	1.03 (0.63)	1.09 (0.73)
Nonsupport leg bent approximately 90 degrees	0.98 (0.87)	0.92 (0.72)
Gallop	4.64 (1.56)	4.98 (1.87)
Arms bent and lifted to waist level at takeoff	1.36 (0.71)	1.33 (0.63)
A step forward with lead foot followed by step with the trailing foot to a position adjacent to or behind the lead foot	1.27 (0.66)	1.38 (0.65)
Brief period when both feet are off the floor	1.24 (0.66)	1.35 (0.67)
Maintains a rhythmic pattern for four consecutive gallops	0.98 (0.71)	1.01 (0.72)
Hop	4.69 (1.55)	5.21 (1.45)
Nonsupport leg swings forward in pendular fashion to produce force	1.05 (0.74)	1.08 (0.76)
Foot of nonsupport leg remains behind body	1.02 (0.70)	1.13 (0.75)
Arms flexed and swing forward to produce force	1.20 (0.71)	1.27 (0.69)
Takes off and lands three consecutive times on preferred foot	1.09 (0.68)	1.12 (0.72)
Takes off and lands three consecutive times on other foot	0.73 (0.81)	0.63 (0.71)
Leap	4.35 (1.87)	3.63 (1.54)
Take off on one foot and land on the opposite foot	0.98 (0.71)	1.17 (0.74)
A period where both feet are off the ground longer than running	1.03 (0.75)	1.36 (0.68)
Forward reach with the arm opposite the lead foot	1.17 (0.73)	1.09 (0.70)
Horizontal Jump	4.69 (1.75)	4.99 (1.67)
Preparatory movement includes flexion of both knees with arms extended behind body	1.23 (0.70)	1.30 (0.72)
Arms extended forcefully forward and upward reaching full extension above the head	1.14 (0.65)	1.12 (0.68)
Take off and land on both feet simultaneously	1.11 (0.70)	1.28 (0.64)
Arms are thrust downward during landing	1.17 (0.70)	1.28 (0.69)
Slide	4.16 (1.56)	5.41 (2.12)
Body turned sideways so shoulders are aligned with the line on the floor	1.12 (0.73)	1.20 (0.69)
A step sideways with lead foot followed by a slide of the trailing foot to a point next to the lead foot	1.16 (0.76)	1.27 (0.70)
A minimum of four continuous step-slides cycles to the right	0.97 (0.70)	1.04 (0.80)
A minimum of four continuous step-slides cycles to the left	0.92 (0.69)	0.88 (0.60)

**Table 3:** Percentage (%) of children who master the skill criterion (locomotor skills).

	3 Years	4 Years	5 Years
	%	%	%
Run			
Arms move in opposition to legs, elbows bent	65.4	45	50
Brief period where both feet are off the ground	65	76	85
Narrow foot placement, landing on heel or toe	73	81	82
Nonsupport leg bent approximately 90 degrees	74.3	81.4	90.2
Gallop			
Arms bent and lifted to waist level at takeoff	21.1	46.1	48
A step forward with lead foot followed by step with the trailing foot to a position adjacent to or behind the lead foot	24.6	57.9	64
Brief period when both feet are off the floor	17.5	46.1	58
Maintains a rhythmic pattern for four consecutive gallops	19.3	23.7	32
Hop			
Nonsupport leg swings forward in pendular fashion to produce force	22.8	44	52
Foot of nonsupport leg remains behind body	33.3	37	40
Arms flexed and swing forward to produce force	31.6	52.6	55
Takes off and lands three consecutive times on preferred foot	17.5	36.8	40
Takes off and lands three consecutive times on other foot	18.2	36.2	43
Leap			
Take off on one foot and land on the opposite foot	22.8	43.3	50
A period where both feet are off the ground longer than running	28.1	47.4	58
Forward reach with the arm opposite the lead foot	17.5	35.5	42
Horizontal Jump			
Preparatory movement includes flexion of both knees with arms extended behind body	37.7	42.2	51.7
Arms extended forcefully forward and upward reaching full extension above the head	17.4	34.8	40.2
Take off and land on both feet simultaneously	32.1	36.5	38
Arms are thrust downward during landing	39.4	43.1	45.6
Slide			
Body turned sideways so shoulders are aligned with the line on the floor	24.8	34.5	41.1
A step sideways with lead foot followed by a slide of the trailing foot to a point next to the lead foot	34.9	39.7	44.8
A minimum of four continuous step-slides cycles to the right	20.2	25	51.7
A minimum of four continuous step-slides cycles to the left	17.2	27	41

**Table 4:** Means (M) and standard deviations (SD) of performance on the TGMD-2—item scores and subtest scores—for boys and girls in object control development.

	Girls	Boys
	M (SD)	M (SD)
Stationary ball	4.35 (2.05)	4.97 (2.10)
Dominant hand grips bat above nondominant hand	1.27 (0.67)	1.39 (0.66)
Nonpreferred side of body faces the imaginary tosser with feet parallel	0.83 (0.71)	1.08 (0.68)
Hip and shoulder rotation during swing	0.80 (0.67)	0.89 (0.68)
Transfer body weight to front foot	0.80 (0.68)	0.88 (0.72)
Bat contacts ball	0.88 (0.72)	0.91 (0.63)
Dribble	2.93 (2.05)	3.24 (2.03)
Contacts ball with one hand about belt level	0.80 (0.69)	0.92 (0.73)
Pushes ball with fingertips	0.70 (0.72)	0.80 (0.68)
Ball contacts surface in front of or to the outside of foot on the preferred side	0.86 (0.73)	0.92 (0.70)
Maintains control of ball for four consecutive bounces without having to move feet to retrieve it	0.55 (0.65)	0.61 (0.69)
Catch	3.08 (1.50)	3.44 (2.12)
Preparation phase where hands are in front of the body and elbows are flexed	1.04 (0.62)	1.15 (0.81)
Arms extended while reaching for the ball as it arrives	1.10 (0.65)	1.29 (0.75)
Ball is caught by hands only	0.94 (0.73)	1.06 (0.65)
Kick	4.39 (1.89)	4.99(2.35)
Rapid continuous approach to the ball	1.30 (0.67)	1.47 (0.62)
An elongated stride or leap immediately prior to ball contact	1.05 (0.65)	1.15 (0.71)
Nonkicking foot placed even with or slightly in back of the ball	0.93 (0.67)	1.10 (0.72)
Kicks ball with instep of preferred foot or toe	1.06 (0.67)	1.28 (0.67)
Overhead throw	3.34 (1.65)	4.05 (2.15)
Windup is initiated with downward movement of hand/arm	1.02 (0.76)	1.24 (0.67)
Rotates hips and shoulders to a point where the nonthrowing side faces the wall	0.77 (0.67)	0.94 (0.75)
Weight is transferred by stepping with the foot opposite the throwing hand	0.79 (0.69)	0.98 (0.73)
Follow-through beyond ball release diagonally across the body toward the nonpreferred side	0.77 (0.71)	0.90 (0.67)
Underhand role	3.83 (1.77)	4.18 (2.17)
Preferred hand swings down and back, reaching behind the trunk while chest faces cones	1.11 (0.65)	1.25 (0.74)
Strides forward with foot opposite the preferred hand toward the cones	0.95 (0.68)	1.07 (0.66)
Bends knees to lower body	1.12 (0.75)	1.04 (0.68)
Releases ball close to the floor so ball does not bounce more than 4 inches high	0.73 (0.76)	0.85 (0.67)

**Table 5:** Percentage (%) of children who master the skill criterion (object control skills).

	3 Years	4 Years	5 Years
	%	%	%
Stationary ball			
Dominant hand grips bat above nondominant hand	24.9	30	33
Nonpreferred side of body faces the imaginary tosser with feet parallel	21.1	23.3	24.1
Hip and shoulder rotation during swing	8.3	17.1	41.1
Transfer body weight to front foot	11	22.4	23
Bat contacts ball	12.8	22	24
Dribble			
Contacts ball with one hand about belt level	12.8	21.6	25.9
Pushes ball with fingertips	4.6	23.3	25
Ball contacts surface in front of or to the outside of foot on the preferred side	11	24.1	27
Maintains control of ball for four consecutive bounces without having to move feet to retrieve it	6.4	10	13
Catch			
Preparation phase where hands are in front of the body and elbows are flexed	7.3	10.1	12
Arms extended while reaching for the ball as it arrives	35.7	46.6	48.2
Ball is caught by hands only	22	23.3	27.6
Kick			
Rapid continuous approach to the ball	45	52.6	55
An elongated stride or leap immediately prior to ball contact	40.4	45	47.2
Nonkicking foot placed even with or slightly in back of the ball	20.2	35.2	37.6
Kicks ball with instep of preferred foot or toe	25.3	34.2	37
Overhead throw			
Windup is initiated with downward movement of hand/arm	30.2	45.7	48.6
Rotates hips and shoulders to a point where the nonthrowing side faces the wall	16.5	25.9	27.3
Weight is transferred by stepping with the foot opposite the throwing hand	15.6	33.6	36
Follow-through beyond ball release diagonally across the body toward the nonpreferred side	13.6	18.1	20
Underhand role			
Preferred hand swings down and back, reaching behind the trunk while chest faces cones	1.8	4.3	5.6
Strides forward with foot opposite the preferred hand toward the cones	35.3	45.7	52
Bends knees to lower body	21.1	25	29
Releases ball close to the floor so ball does not bounce more than 4 inches high	14.7	16.1	22.4

**Table 6:** Percentage (%) of Spanish children compared to American children.

	Spanish Children			American Children		
Skills	3	4	5	3	4	5
Run	30.5	45	50	40	54	54
Gallop	6.4	6.9	20.7	5	13	24
Hop	1	2.6	10.2	2	14	31
Leap	3.1	4	5.1	7	21	14
Horizontal jump	6.4	12.9	13.5	10	11	17
Slide	3.7	7.8	8.65	11	29	34
Stationary ball	1	6	7.8	13	12.5	17
Dribble	1.8	2.1	3	2	5	9
Catch	2.5	11.2	13.2	1	45	15
Kick	5.2	8.4	12.1	6	10	23
Overhead throw	4.6	8.6	10.2	6	12	18
Underhand roll	1.8	4.3	7.2	1	6	11

**Table 7:** Descriptive statistics and Mann–Whitney U test, taking gender into account.

	Age (Years)	Girls			Boys			U	
		M	Md	SD	M	Md	SD	U	Z
Locomotor development	3	20.38	21	7.07	21	22	7.30	1325.5	−0.504 *
	4	22.41	22	6.70	25.83	25	8.30	1242	−2.31 *
	5	26.21	25	9.52	27.36	26	9.34	394.5	−0.283
Object control development	3	17.81	16.50	7.30	21.34	21	8.99	1053	−2.20 *
	4	22.96	22	9.48	27.26	28	10.53	1248	−2.28 *
	5	21.45	18	10.78	26.88	24	10.66	282.50	−2.04 *

M = Mean, Md = Mean Deviation, SD = Standard Deviation, * *p* < 0.05.

**Table 8:** Descriptive statistics and Mann–Whitney U test, taking into account being or not an only child.

		Yes			No		U Mann Withney	
	M	Md	SD	M	Md	SD	U	Z
Locomotor development	23.62	24	8.29	23.66	24	8.44	10,812	−0.062
Object control development	22.43	22.50	10.13	23.44	23	10.05	10,209.5	−0.879

M = Mean, Md = Mean Deviation, SD = Standard Deviation.

**Table 9:** Percentage (%) of children who demonstrate mastery of skills, taking into account extracurricular activities.

	Extracurricular Physical Activities	
Skills	No	Yes
Run	60.5	76.6
Gallop	40.3	56.5
Hop	26.5	30.6
Leap	17.6	20.1
Horizontal jump	7.9	13.2
Slide	15.1	26.7
Stationary ball	2.4	5.2
Dribble	1	2.3
Catch	4.8	12.1
Kick	9.5	15.5
Overhead throw	4	7.5
Underhand roll	2.4	4.6

**Table 10:** Descriptive statistics and Mann–Whitney U test, taking into account extracurricular activity.

		Yes			No		U Mann Withney	
	M	Md	SD	M	Md	SD	U	Z
Locomotor development	24.33	25	8.10	22.58	23	8.66	7831.5	−4.06
Object control development	25.46	26	10.44	19.62	17	8.57	7124.5	−5.02 *

M = Mean, Md = Mean Deviation, SD = Standard Deviation, * *p* < 0.05.

**Table 11:** Descriptive statistics and Mann–Whitney U test, taking into account prematurity.

		No			Yes		U Mann Whitney	
	M	Md	SD	M	MD	SD	U	Z
Locomotor development	23.91	24	8.44	20.48	20	6.83	2616	−0.818
Object control development	23.24	23	10.23	20.81	20	7.47	2332	−1.52

M = Mean, Md = Mean Deviation, SD = Standard Deviation.

## Data Availability

Data available on request due to restrictions (private information). The data presented in this study are available on request from the corresponding author. The data are not publicly available due to ethical principles.
